# Methotrexate‐Associated Lymphoproliferative Disorders Characterized by a T‐Cell Phenotype With Lung and Psoas Involvement: A Case Report

**DOI:** 10.1155/carm/7668419

**Published:** 2026-01-28

**Authors:** Katsunori Arai, Hirokazu Tokuyasu, Yuriko Sueda, Hiromitsu Sakai, Hirara Watase, Mikako Yoshioka, Yoshiki Naritomi, Chika Esumi, Akira Yamasaki

**Affiliations:** ^1^ Respiratory Medicine Division, Matsue Red Cross Hospital, Matsue, Shimane, Japan, jrc.or.jp; ^2^ Pathology Division, Matsue Red Cross Hospital, Matsue, Shimane, Japan, jrc.or.jp; ^3^ Department of Multidisciplinary Internal Medicine, Division of Respiratory Medicine and Rheumatology, School of Medicine, Faculty of Medicine, Tottori University, Yonago, Tottori, Japan, tottori-u.ac.jp

**Keywords:** methotrexate-associated lymphoproliferative disorder, peripheral T-cell lymphoma, psoas muscle involvement, remission, rheumatoid arthritis

## Abstract

Methotrexate (MTX) can cause MTX‐associated lymphoproliferative disorders (MTX‐LPDs) characterized by a T‐cell phenotype. Herein, we report an extremely rare case of MTX‐LPD with peripheral T‐cell lymphoma (PTCL) phenotype and lung and skeletal muscle involvement. A 66‐year‐old man who had received MTX for 8 years for rheumatoid arthritis was admitted for right groin pain. A computed tomography scan revealed masses in the right middle lobe and right psoas muscle and multiple nodules in both lung fields. Pathological and immunohistochemical examinations of a lung nodule and psoas muscle tumor revealed PTCL. Remission was achieved soon after discontinuing MTX.

## 1. Introduction

Lymphoproliferative disorders (LPDs) arise from the excessive growth of lymphatic system cells. Medications, such as antitumor necrosis factor inhibitors, can induce LPDs, which the World Health Organization classifies as other iatrogenic immunodeficiency–associated LPDs; this type is one of the four subgroups associated with immunodeficiency [1]. Autoimmune disorder medications, such as methotrexate (MTX) for rheumatoid arthritis (RA), can also induce LPDs, known as MTX‐associated LPDs (MTX‐LPDs). The most common histopathological finding of MTX‐LPDs is diffuse large B‐cell lymphoma, occurring in 52.0% of patients, whereas T‐cell lymphoma is relatively rare, occurring in only 3.9% of patients [2]. Skeletal muscle involvement is unusual for extranodal occurrence of any lymphoma, particularly T‐cell lymphoma [3, 4].

Herein, we report an extremely rare case of MTX‐LPD characterized by the peripheral T‐cell lymphoma (PTCL), not otherwise specified (PTCL‐NOS) phenotype with lung and skeletal muscle involvement. Moreover, complete remission was achieved after discontinuing MTX.

## 2. Case Presentation

A 66‐year‐old man who had never smoked presented with right groin pain that persisted for 3 weeks. The patient was diagnosed with RA at 58 years of age, for which he had been taking 8 mg/week of MTX for 8 years; he did not take any other anti‐RA medications. Physical examination revealed no abnormalities, and superficial lymphadenopathy was not observed. The patient’s white blood cell count was 6.7 × 10^9^/L with 72.5% neutrophils, 10.0% lymphocytes, 11.5% monocytes, 5.0% eosinophils, and 1.0% basophils. The C‐reactive protein and serum soluble interleukin–2 receptor (sIL2R) levels were elevated to 2.19 mg/dL (normal range: 0–0.3 mg/dL) and 1264 U/mL (normal range: 127–582 U/mL), respectively. A serum antibody test for human T‐cell leukemia virus–1 was negative. Computed tomography (CT) revealed a mass in the right middle lobe, multiple nodules in both lung fields, and a mass in the right psoas muscle (Figure 1). Bronchoscopy of the right middle lobe tumor was performed (Figure 1(a)); however, no diagnosis was made. Therefore, CT‐guided needle biopsy with a Monopty 18‐gauge needle (Bard Covington GA, U.S.) of a left pulmonary nodule in contact with the pleura, which was considered to be easily accessible for tissue sampling, was performed (Figure 1(b)). Two biopsies were performed with 1% xylocaine injection for anesthesia. The tissues collected were two pieces measuring 0.7 × 3.5 mm. A mild pneumothorax occurred as a complication of the examination, but no other complication was noted. To collect a larger sample volume, a surgical biopsy of the right psoas muscle was performed (Figures 1(c) and 1(d)) and yielded a specimen measuring 12 × 32 mm. A pathological examination of the left pulmonary nodule and the right psoas muscle was conducted. Hematoxylin and eosin and immunohistochemical staining results were consistent with those of PTCL‐NOS (Figure 2). In situ hybridization revealed that the lymphocytes were negative for Epstein–Barr virus (EBV)–encoded small RNA. Clonal rearrangement of the T‐cell receptor beta chain was detected; however, immunoglobulin heavy chains were not detected in the lung nodule by polymerase chain reaction. MTX‐LPD of the PTCL‐NOS phenotype was diagnosed, and MTX was discontinued. Two weeks later, the patient became asymptomatic, and CT revealed shrinkage of the pulmonary and psoas tumors. After 6 months, the patient’s sIL‐2R levels normalized, and CT revealed resolution of the lesions (Figure 3). The patient remained healthy with no recurrence 2 years after discontinuing MTX.

**Figure FIGURE 1:**
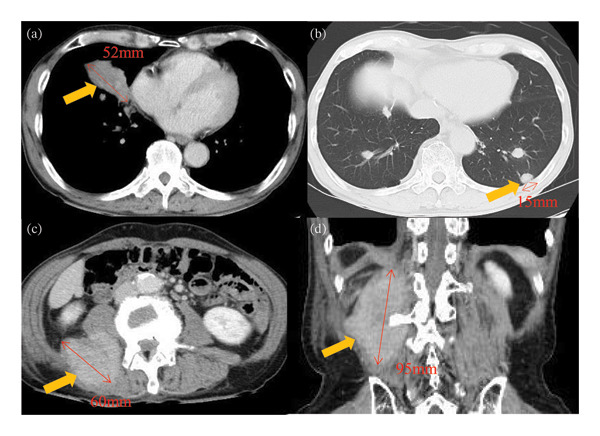
CT images obtained upon admission. (a) CE chest CT reveals a mass in the right middle lobe with a tumor major axis of 52 mm (arrow). (b) Chest CT reveals multiple nodules in both lung fields. The major axis of the tumor in contact with the pleura measured 15 mm (arrow). (c) CE axial abdominal CT reveals a mass in the right psoas muscle with the tumor’s major axis measuring 60 mm (arrow). (d) CE coronal abdominal CT reveals a mass in the right psoas with the tumor’s major axis measuring 95 mm (arrow). CE: contrast‐enhanced; CT: computed tomography.

**Figure FIGURE 2:**
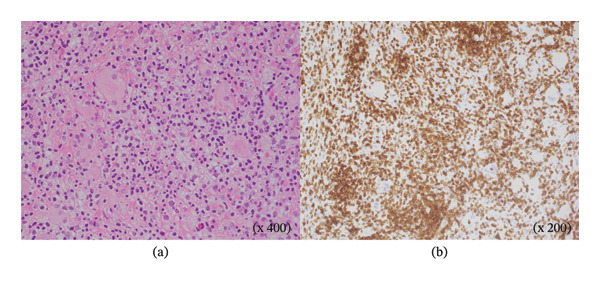
Microscopic histopathology of a biopsy specimen obtained from a mass in the right psoas muscle. (a) Hematoxylin‐eosin staining reveals dense proliferation of small lymphocytes. (b) Immunostaining reveals CD3‐positive lymphocyte infiltration. CD3: cluster of differentiation 3.

**Figure FIGURE 3:**
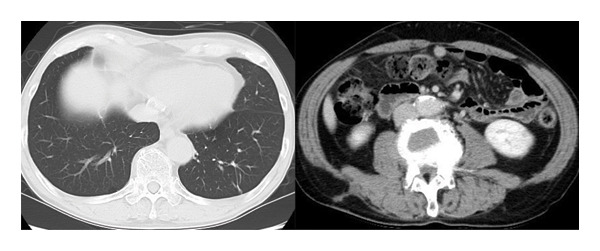
Four months after discontinuing methotrexate, CT reveals spontaneous resolution of the lung and psoas lesions.

## 3. Discussion

In this case, the pathological phenotype of the MTX‐LPD was PTCL‐NOS. T‐Cell lymphoma and PTCL‐NOS account for 6.3% and 1.7% of mature noncutaneous lymphomas, respectively [5]. Tanaka et al. evaluated 986 patients with RA, reporting that the cumulative incidence of MTX‐LPD 5 and 10 years after MTX initiation was 1.3% and 4.7%, respectively [6]. MTX‐LPD with a T‐cell phenotype is relatively rare; for example, Ichikawa et al. reported 4 cases (3.9%) of T‐cell lymphoma after examining 102 patients with MTX‐LPD [2]. Satou et al. also analyzed 28 patients with MTX‐LPD and T‐cell phenotypes [7]. In their study, the tumors were divided into three main types based on histological and immunohistochemical characteristics: angioimmunoblastic T‐cell lymphoma (*n* = 19, 67.9%), PTCL‐NOS (*n* = 6, 10.7%), and CD8+ cytotoxic T‐cell lymphoma (*n* = 3, 21.4%). The results showed that 27 patients (96%) had lymphadenopathy, including 11 with extranodal involvement, and the most frequent sites of extranodal involvement were the skin (*n* = 3), spleen (*n* = 3), liver (*n* = 2), and bone marrow (*n* = 2) [7]. Moreover, relatively few reports of MTX‐LPD with a T‐cell phenotype and pulmonary involvement exist [8, 9], as seen in this case.

Lymphomatous muscle involvement reportedly occurs in 0.3% of Hodgkin’s lymphoma and 1.1% of non‐Hodgkin’s lymphoma cases [10], most often due to hematogenous or lymphatic spread or contiguous spread from adjacent involvement of the lymph nodes or bone. Reports on the muscle regions affected by T‐cell lymphoma are limited but indicate that the muscles in the extremities are the most affected [4]. Several cases of B‐cell lymphomatous involvement of the psoas muscle have been reported [11, 12]. However, we found only one reported case of T‐cell lymphoma involving the psoas muscle [4].

In the present case, the patient showed no evidence of EBV infection. Latent EBV infection is suspected to contribute to the development of MTX‐LPD [2]. EBV can infect B cells via CD21, which is also detected in normal T cells [13]. Satou et al. reported only one case in which EBV‐encoded small RNA was expressed in T‐cell tumor cells, but scattered EBV‐infected B cells were detected in 24 cases (89%), two of which were PTCL‐NOS cases [7]. These findings suggest that an immunodeficient state reactivates EBV. EBV may also be involved in the oncogenesis of MTX‐LPD characterized by a T‐cell phenotype.

Among patients with RA treated with MTX, survival does not differ between those who do and do not develop LPD [6]; thus, the prognosis for MTX‐LPD appears to be good. Approximately 40%–60% of patients with MTX‐LPD achieved remission after discontinuing MTX [14, 15]. The frequency of remission varies according to the histological subtype of LPD. Spontaneous remission after MTX discontinuation is reportedly higher in T‐cell than in B‐cell phenotype MTX‐LPDs (77% vs. fifty‐three percent). However, one study reported lower survival and progression‐free survival rates for patients with MTX‐LPD and a T‐cell phenotype than for those with a B‐cell phenotype [7]. In that study, four of the 26 patients with MTX‐LPD characterized by a T‐cell phenotype and spontaneous remission after MTX treatment experienced relapse or progression. In such cases, chemotherapy is administered based on the histological type. However, the clinical outcome for most patients with T‐cell lymphomas is poor on standard therapies [16]. Even if remission is achieved, relapse can still occur; consequently, careful observation is required. In our case, the lesions rapidly shrunk after MTX discontinuation, and recurrence did not occur after 2 years.

In conclusion, we present an extremely rare case of MTX‐LPD characterized by a T‐cell phenotype with lung and psoas muscle involvement. Although complete remission was achieved by discontinuing MTX, MTX‐LPD characterized by a T‐cell phenotype is considered to have a poor prognosis if relapses occur, and strict follow‐up is required.

## Funding

No funding was received for this work.

## Consent

Written informed consent for publication was obtained from the patient.

## Conflicts of Interest

The authors declare no conflicts of interest.

## Data Availability

The data used to support the findings of this study are available from the corresponding author upon reasonable request.
